# Supplementary material for: Exploration of the Binding Mechanism of Cyclic Dinucleotide Analogs to Stimulating Factor Proteins and the Implications for Subsequent Analog Drug Design
Source: Biomolecules. 2024 Mar 14;14(3):350. doi: 10.3390/biom14030350 (PMC10967758; doi:10.3390/biom14030350)
Supplement: Supplementary file 1 [file biomolecules-14-00350-s001.zip › biomolecules-2913002-supplementary.pdf]

Exploration of the binding mechanism of cyclic dinucleotide analogs to STING proteins and the implications for subsequent analog drug design

Figure S1. The confidence index of AlphaFold2 modeling for hSTING-WT. Subsequent modeling will use the same parameter.

(A)

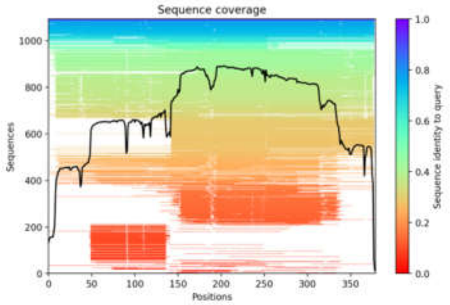

(B)

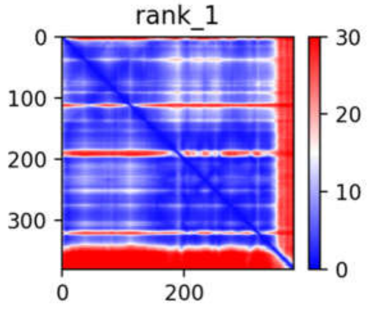

(C)

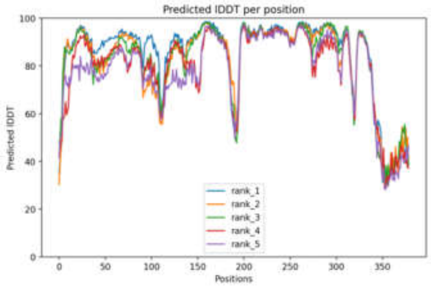

(A) Sequence coverage of hSTING-WT in the modeling, (B) Plots of the model quality for rank1, (C) Predicted IDDT value per positions in hSTING-WT for

rank1 to rank5.

Table S1. Score list of the molecular docking for hSTING-WT-cGAMP

Mode	Affinity (kcal/mol)	Dist from best mode RMSD l.b.	Dist from best mode RMSD u.b.
**1**	-7.5	0.000	0.000
**2**	-7.4	6.623	9.256
**3**	-7.1	4.451	9.728
**4**	-7.0	4.766	7.179
**5**	-7.0	6.961	10.915
**6**	-7.0	3.985	9.313
**7**	-6.9	3.214	4.603
**8**	-6.9	2.667	9.556
**9**	-6.5	3.347	9.743

Table S2. Score list of the molecular docking for hSTING-WT-c-AMP-CMP

Mode	Affinity (kcal/mol)	Dist from best mode RMSD l.b.	Dist from best mode RMSD u.b.
**1**	-7.6	0.000	0.000
**2**	-7.1	2.471	2.903
**3**	-6.9	2.321	2.583
**4**	-6.8	1.748	10.834
**5**	-6.7	4.556	6.795
**6**	-6.7	3.302	3.993
**7**	-6.6	7.448	9.899
**8**	-6.1	5.896	8.257
**9**	-6.1	3.367	10.568

Table S3. Score list of the molecular docking for hSTING-WT-2′-F-c-di-GMP

Mode	Affinity (kcal/mol)	Dist from best mode	
		RMSD l.b.	RMSD u.b.
**1**	-6.6	0.000	0.000
**2**	-6.6	0.123	5.811
**3**	-6.3	6.303	10.320
**4**	-6.1	3.555	6.253
**5**	-6.1	3.556	7.288
**6**	-5.8	1.869	2.246
**7**	-5.8	3.108	6.018
**8**	-5.1	3.323	6.247
**9**	-5.1	6.274	9.745

Table S4. Score list of the molecular docking for hSTING-WT-Rp, Rp-2',3-c-di-AMI.

Mode	Affinity (kcal/mol)	Dist from best mode	
		RMSD l.b.	RMSD u.b.
**1**	-6.9	0.000	0.000
**2**	-6.6	3.379	10.938
**3**	-6.3	2.586	10.670
**4**	-6.3	3.285	4.437
**5**	-6.0	2.709	10.108
**6**	-6.0	2.147	2.859
**7**	-5.9	6.144	10.601
**8**	-5.7	7.035	10.513
**9**	-5.5	6.470	10.337

Table S5. Score list of the molecular docking for hSTING-WT-CDG^{SF}.

Mode	Affinity (kcal/mol)	Dist from best mode	
		RMSD l.b.	RMSD u.b.
**1**	-6.9	0.000	0.000
**2**	-6.9	1.918	2.244
**3**	-6.7	1.991	5.945
**4**	-6.5	2.741	6.351
**5**	-6.4	8.027	11.134
**6**	-6.4	6.797	9.635
**7**	-6.0	7.249	11.110
**8**	-6.0	7.758	10.530
**9**	-5.7	4.795	7.616

Table S6. Score list of the molecular docking for hSTING-WT-MK-1454.

Mode	Affinity (kcal/mol)	Dist from best mode	
		RMSD l.b.	RMSD u.b.
1	-7.2	0.000	0.000
2	-7.1	8.672	12.592
3	-7.0	3.876	5.324
4	-6.9	6.059	8.816
5	-6.9	8.817	12.354
6	-6.7	3.617	4.625
7	-6.6	1.898	2.593
8	-6.6	7.327	10.399
9	-6.3	6.709	11.522

Table S7. Score list of the molecular docking for hSTING-WT-ribo/xylo-19.

Mode	Affinity (kcal/mol)	Dist from best mode	
		RMSD l.b.	RMSD u.b.
1	-7.3	0.000	0.000
2	-7.2	7.253	9.685
3	-6.8	6.233	8.306
4	-6.5	5.048	7.716
5	-6.3	3.155	9.537
6	-6.3	8.855	13.000
7	-6.3	4.458	9.281
8	-6.1	1.603	2.016
9	-6.1	2.130	8.996

Table S8. Score list of the molecular docking for hSTING-WT-cyclic PNAs.

Mode	Affinity (kcal/mol)	Dist from best mode	
		RMSD l.b.	RMSD u.b.
1	-8.1	0.000	0.000
2	-8.0	3.341	10.493
3	-7.9	2.125	3.129
4	-7.9	1.948	2.341
5	-7.6	1.386	2.445
6	-7.4	1.972	2.806
7	-7.3	3.543	7.667
8	-7.3	3.991	9.516
9	-7.3	4.986	8.361

Table S9. Score list of the molecular docking for hSTING-WT-DMXAA.

Mode	Affinity (kcal/mol)	Dist from best mode	
		RMSD l.b.	RMSD u.b.
1	-6.9	0.000	0.000
2	-6.9	1.482	4.837
3	-6.5	2.149	5.394
4	-6.3	3.245	6.084
5	-6.1	1.829	4.450
6	-6.0	2.805	4.413
7	-6.0	2.183	3.663
8	-5.8	1.982	4.983
9	-5.8	1.880	4.114

Table S10. Score list of the molecular docking for hSTING-WT-α-Mangostin.

Mode	Affinity (kcal/mol)	Dist from best mode	
		RMSD l.b.	RMSD u.b.
1	-7.6	0.000	0.000
2	-7.5	2.651	4.783
3	-7.5	1.792	2.633
4	-7.2	2.474	8.784
5	-6.9	2.170	7.510
6	-6.8	2.435	4.568
7	-6.6	4.787	10.151
8	-6.5	2.570	8.700
9	-6.2	2.768	8.428

Table S11. Score list of the molecular docking for hSTING-WT-di-ABZI.

Mode	Affinity (kcal/mol)	Dist from best mode	
		RMSD l.b.	RMSD u.b.
1	-6.3	0.000	0.000
2	-6.2	6.923	10.149
3	-6.0	2.727	5.783
4	-5.9	2.212	4.903
5	-5.9	1.849	2.733
6	-5.8	1.290	2.162
7	-5.6	2.145	5.640
8	-5.6	7.031	11.036
9	-5.6	2.404	4.849

Table S12. Score list of the molecular docking for hSTING-WT-D61.

Mode	Affinity (kcal/mol)	Dist from best mode	
		RMSD l.b.	RMSD u.b.
1	-5.7	0.000	0.000
2	-5.4	3.991	8.042
3	-5.4	4.191	8.061
4	-5.4	4.158	6.446
5	-4.1	1.643	2.156
6	-4.1	5.110	9.799
7	-3.9	5.717	10.161
8	-3.4	2.287	3.220
9	-3.2	4.469	8.414

Table S13. Score list of the molecular docking for hSTING-R232H-cGAMP.

Mode	Affinity (kcal/mol)	Dist from best mode	
		RMSD l.b.	RMSD u.b.
1	-7.8	0.000	0.000
2	-7.6	1.611	1.796
3	-7.4	7.470	11.504
4	-7.4	4.570	9.624
5	-7.4	4.325	9.973
6	-7.2	4.846	9.781
7	-7.1	2.284	10.033
8	-7.1	3.699	9.780
9	-7.1	2.186	9.845

Table S14. Score list of the molecular docking for hSTING-R232H-c-AMP-CMP.

Mode	Affinity (kcal/mol)	Dist from best mode	
		RMSD l.b.	RMSD u.b.
1	-7.4	0.000	0.000
2	-7.3	1.794	2.288
3	-7.0	8.115	10.228
4	-7.0	1.768	10.831
5	-6.7	8.084	10.802
6	-6.6	1.366	10.747
7	-6.6	7.491	9.989
8	-6.6	4.478	6.699
9	-6.5	5.461	10.654

Table S15. Score list of the molecular docking for hSTING-R232H-2′-F-c-di-GMP.

Mode	Affinity (kcal/mol)	Dist from best mode	
		**RMSD l.b.**	**RMSD u.b.**
**1**	-6.7	0.000	0.000
**2**	-6.6	0.192	5.811
**3**	-6.4	2.648	3.715
**4**	-6.2	3.604	5.818
**5**	-6.2	3.459	6.099
**6**	-6.2	3.458	7.263
**7**	-6.1	6.835	11.202
**8**	-6.0	6.772	10.810
**9**	-6.0	13.772	16.244

Table S16. Score list of the molecular docking for hSTING-R232H-Rp, Rp-2',3-c-di-AMI.

Mode	Affinity (kcal/mol)	Dist from best mode	
		**RMSD l.b.**	**RMSD u.b.**
**1**	-6.9	0.000	0.000
**2**	-6.7	3.681	10.581
**3**	-6.6	3.552	11.049
**4**	-6.5	8.601	10.742
**5**	-6.4	2.725	10.700
**6**	-6.0	6.117	10.531
**7**	-6.0	3.009	10.321
**8**	-5.8	1.739	2.165
**9**	-5.6	6.581	10.608

Table S17. Score list of the molecular docking for hSTING-R232H-CDG^{SF}.

Mode	Affinity (kcal/mol)	Dist from best mode	
		RMSD l.b.	RMSD u.b.
1	-7.2	0.000	0.000
2	-7.1	2.538	5.560
3	-7.0	2.527	3.771
4	-6.9	3.759	6.499
5	-6.7	2.946	4.820
6	-6.7	4.313	7.293
7	-6.7	2.403	3.707
8	-6.6	4.130	7.046
9	-6.4	5.754	8.839

Table S18. Score list of the molecular docking for hSTING-R232H-MK-1454.

Mode	Affinity (kcal/mol)	Dist from best mode	
		RMSD l.b.	RMSD u.b.
1	-7.2	0.000	0.000
2	-7.2	4.307	5.856
3	-7.0	4.071	10.116
4	-6.9	6.080	7.565
5	-6.8	5.935	9.752
6	-6.6	5.020	6.743
7	-6.6	4.922	6.540
8	-6.3	6.136	7.811
9	-6.3	5.658	8.872

Table S19. Score list of the molecular docking for hSTING-R232H-ribo/xylo-19.

Mode	Affinity (kcal/mol)	Dist from best mode	
		RMSD l.b.	RMSD u.b.
1	-7.9	0.000	0.000
2	-7.3	2.847	4.063
3	-7.3	2.885	8.778
4	-7.3	7.240	9.775
5	-7.3	2.445	9.005
6	-7.0	2.555	8.932
7	-6.7	3.369	6.458
8	-6.7	4.072	6.764
9	-6.5	3.640	6.060

Table S20. Score list of the molecular docking for hSTING-R232H-cyclic PNAs .

Mode	Affinity (kcal/mol)	Dist from best mode	
		RMSD l.b.	RMSD u.b.
1	-8.3	0.000	0.000
2	-8.0	3.170	10.930
3	-8.0	3.193	10.642
4	-7.7	3.225	10.714
5	-7.7	1.909	2.631
6	-7.6	2.936	10.837
7	-7.3	3.099	11.326
8	-7.3	3.177	9.855
9	-7.2	1.828	2.588

Table S21. Score list of the molecular docking for hSTING-R232H-DMXAA.

Mode	Affinity (kcal/mol)	Dist from best mode	
		RMSD l.b.	RMSD u.b.
1	-6.9	0.000	0.000
2	-6.8	1.475	4.850
3	-6.6	2.205	5.522
4	-6.1	2.901	4.433
5	-6.1	2.233	6.588
6	-6.0	3.616	5.352
7	-5.9	3.023	5.617
8	-5.8	2.006	4.483
9	-5.6	11.993	14.279

Table S22. Score list of the molecular docking for hSTING-R232H-α-Mangostin.

Mode	Affinity (kcal/mol)	Dist from best mode	
		RMSD l.b.	RMSD u.b.
1	-7.6	0.000	0.000
2	-7.5	2.218	4.102
3	-7.2	1.426	2.082
4	-7.0	2.315	4.031
5	-6.8	2.093	8.618
6	-6.6	2.305	8.417
7	-6.3	2.311	3.572
8	-6.1	2.480	8.276
9	-6.0	2.732	4.554

Table S23. Score list of the molecular docking for hSTING-R232H-di-ABZI.

Mode	Affinity (kcal/mol)	Dist from best mode	
		RMSD l.b.	RMSD u.b.
1	-6.8	0.000	0.000
2	-6.8	1.739	2.379
3	-6.7	2.170	6.325
4	-6.3	2.439	5.127
5	-6.3	4.051	8.703
6	-6.1	2.284	6.988
7	-6.1	1.893	6.974
8	-6.0	1.761	4.659
9	-6.0	1.892	2.845

Table S24. Score list of the molecular docking for hSTING-R232H-D61.

Mode	Affinity (kcal/mol)	Dist from best mode	
		RMSD l.b.	RMSD u.b.
1	-6.0	0.000	0.000
2	-6.0	2.052	5.769
3	-5.9	3.595	4.798
4	-5.8	3.436	4.223
5	-5.8	4.321	7.581
6	-5.8	3.326	7.061
7	-5.8	3.154	7.264
8	-5.6	2.964	7.414
9	-5.4	2.683	7.071

Table S25. Score list of the molecular docking for hSTING-R293Q-cGAMP.

Mode	Affinity (kcal/mol)	Dist from best mode	
		RMSD l.b.	RMSD u.b.
1	-7.5	0.000	0.000
2	-7.5	6.603	9.192
3	-7.1	6.966	10.993
4	-7.0	3.970	9.318
5	-6.9	3.205	4.598
6	-6.8	8.082	10.084
7	-6.7	5.164	7.413
8	-6.6	7.847	9.923
9	-6.6	2.663	9.633

Table S26. Score list of the molecular docking for hSTING-R293Q-c-AMP-CMP.

Mode	Affinity (kcal/mol)	Dist from best mode RMSD l.b.	RMSD u.b.
**1**	-7.5	0.000	0.000
**2**	-7.1	8.091	10.218
**3**	-7.1	2.474	2.901
**4**	-6.8	2.236	2.478
**5**	-6.8	8.112	10.781
**6**	-6.8	1.781	10.825
**7**	-6.7	4.545	6.790
**8**	-6.6	8.062	12.049
**9**	-6.3	1.516	10.764

Table S27. Score list of the molecular docking for hSTING-R293Q-2′-F-c-di-GMP.

Mode	Affinity (kcal/mol)	Dist from best mode RMSD l.b.	RMSD u.b.
**1**	-6.6	0.000	0.000
**2**	-6.6	0.116	5.808
**3**	-6.3	6.294	10.310
**4**	-6.3	6.293	10.507
**5**	-6.1	3.552	6.249
**6**	-6.1	3.552	7.285
**7**	-5.9	1.857	6.001
**8**	-5.8	1.868	2.240
**9**	-5.8	3.107	6.013

Table S28. Score list of the molecular docking for hSTING-R293Q-Rp, Rp-2',3-c-di-AMI.

Mode	Affinity (kcal/mol)	Dist from best mode	
		RMSD l.b.	RMSD u.b.
**1**	-6.9	0.000	0.000
**2**	-6.4	2.722	10.709
**3**	-5.9	6.211	10.613
**4**	-5.9	2.046	2.684
**5**	-5.9	3.443	10.712
**6**	-5.6	13.467	17.239
**7**	-5.6	3.291	10.499
**8**	-5.5	6.436	10.326
**9**	-5.5	4.852	10.125

Table S29. Score list of the molecular docking for hSTING-R293Q-CDG^{SF}.

Mode	Affinity (kcal/mol)	Dist from best mode	
		RMSD l.b.	RMSD u.b.
**1**	-7.4	0.000	0.000
**2**	-7.4	9.442	12.088
**3**	-7.3	2.600	3.927
**4**	-7.3	8.141	10.601
**5**	-7.1	8.908	11.886
**6**	-6.9	2.352	3.658
**7**	-6.9	2.866	4.747
**8**	-6.8	0.943	1.961
**9**	-6.2	2.783	5.700

Table S30. Score list of the molecular docking for hSTING-R293Q-MK-1454.

Mode	Affinity (kcal/mol)	Dist from best mode	
		RMSD l.b.	RMSD u.b.
1	-7.1	0.000	0.000
2	-7.1	8.533	12.490
3	-6.9	6.008	8.693
4	-6.9	8.671	12.244
5	-6.7	5.689	11.169
6	-6.6	1.869	2.578
7	-6.5	6.586	8.793
8	-6.3	7.170	11.753
9	-6.3	7.631	11.760

Table S31. Score list of the molecular docking for hSTING-R293Q-ribo/xylo-19.

Mode	Affinity (kcal/mol)	Dist from best mode	
		RMSD l.b.	RMSD u.b.
1	-7.3	0.000	0.000
2	-7.2	7.268	9.703
3	-6.6	10.269	12.852
4	-6.5	5.060	7.723
5	-6.5	5.734	8.634
6	-6.5	1.718	2.113
7	-6.4	9.726	12.986
8	-6.3	8.633	11.938
9	-6.3	3.163	9.539

Table S32. Score list of the molecular docking for hSTING-R293Q-cyclic PNAs .

Mode	Affinity (kcal/mol)	Dist from best mode	
		RMSD l.b.	RMSD u.b.
1	-8.2	0.000	0.000
2	-8.2	2.999	10.099
3	-8.0	3.295	10.473
4	-7.9	1.917	2.311
5	-7.9	2.110	3.058
6	-7.9	2.938	9.785
7	-7.6	1.380	2.437
8	-7.6	2.338	9.745
9	-7.3	4.129	9.526

Table S33. Score list of the molecular docking for hSTING-R293Q-DMXAA.

Mode	Affinity (kcal/mol)	Dist from best mode RMSD l.b.	RMSD u.b.
1	-6.9	0.000	0.000
2	-6.7	1.483	4.821
3	-6.6	2.177	5.499
4	-6.3	1.678	5.716
5	-6.2	2.830	4.386
6	-6.1	2.259	6.584
7	-6.1	2.082	5.043
8	-5.9	2.132	3.776
9	-5.4	4.135	6.026

Table S34. Score list of the molecular docking for hSTING-R293Q-α-Mangostin.

Mode	Affinity (kcal/mol)	Dist from best mode RMSD l.b.	RMSD u.b.
1	-7.3	0.000	0.000
2	-7.2	3.119	6.015
3	-7.2	2.631	4.811
4	-6.9	1.739	7.683
5	-6.7	1.365	1.653
6	-6.4	3.128	5.859
7	-6.3	2.881	8.361
8	-6.1	4.990	8.224
9	-5.7	2.202	4.162

Table S35. Score list of the molecular docking for hSTING-R293Q-di-ABZI.

Mode	Affinity (kcal/mol)	Dist from best mode RMSD l.b.	RMSD u.b.
1	-6.4	0.000	0.000
2	-6.4	0.177	7.522
3	-6.3	6.701	11.480
4	-6.0	6.639	10.153
5	-6.0	2.710	5.832
6	-5.9	2.278	8.749
7	-5.9	2.054	4.752
8	-5.7	2.520	6.374
9	-5.7	2.130	7.624

Table S36. Score list of the molecular docking for hSTING-R293Q-D61.

**Mode**	**Affinity (kcal/mol)**	**Dist from best mode**	
		**RMSD l.b.**	**RMSD u.b.**
**1**	-6.0	0.000	0.000
**2**	-6.0	3.618	4.878
**3**	-5.8	3.081	7.467
**4**	-5.6	4.321	7.471
**5**	-5.6	2.402	4.144
**6**	-5.6	2.335	4.653
**7**	-5.5	1.893	5.649
**8**	-5.4	4.231	5.603
**9**	-5.3	2.160	6.537

Table S37. Score list of the molecular docking for hSTING-AQ-cGAMP.

**Mode**	**Affinity (kcal/mol)**	**Dist from best mode**	
		**RMSD l.b.**	**RMSD u.b.**
**1**	-7.7	0.000	0.000
**2**	-7.6	1.637	1.828
**3**	-7.4	4.558	9.663
**4**	-7.3	4.386	9.907
**5**	-7.2	4.032	9.296
**6**	-7.1	2.218	10.026
**7**	-7.1	3.627	9.771
**8**	-7.1	2.184	9.853
**9**	-7.0	4.803	9.838

Table S38. Score list of the molecular docking for hSTING-AQ-c-AMP-CMP.

**Mode**	**Affinity (kcal/mol)**	**Dist from best mode**	
		**RMSD l.b.**	**RMSD u.b.**
**1**	-7.5	0.000	0.000
**2**	-7.3	1.804	2.316
**3**	-7.2	1.160	1.386
**4**	-6.9	1.786	10.824
**5**	-6.6	4.490	6.704
**6**	-6.4	7.424	9.870
**7**	-6.2	5.627	10.600
**8**	-6.1	2.892	3.440
**9**	-6.0	5.751	8.034

Table S39. Score list of the molecular docking for hSTING-AQ-2′-F-c-di-GMP.

Mode	Affinity (kcal/mol)	Dist from best mode	
		RMSD l.b.	RMSD u.b.
1	-6.7	0.000	0.000
2	-6.6	0.199	5.812
3	-6.4	2.642	5.512
4	-6.4	2.632	3.723
5	-6.2	6.733	10.994
6	-6.2	3.460	7.264
7	-6.2	3.458	6.096
8	-6.1	6.747	10.779
9	-6.1	14.079	16.602

Table S40. Score list of the molecular docking for hSTING-AQ-Rp, Rp-2',3-c-di-AMI.

Mode	Affinity (kcal/mol)	Dist from best mode	
		RMSD l.b.	RMSD u.b.
1	-6.9	0.000	0.000
2	-6.7	3.681	10.582
3	-6.5	8.593	10.731
4	-6.4	2.723	10.708
5	-6.0	3.065	10.346
6	-6.0	6.146	10.573
7	-5.9	6.443	11.335
8	-5.8	9.078	13.485
9	-5.7	6.585	10.306

Table S41. Score list of the molecular docking for hSTING-AQ-CDG^{SF}.

Mode	Affinity (kcal/mol)	Dist from best mode	
		RMSD l.b.	RMSD u.b.
**1**	-7.3	0.000	0.000
**2**	-7.1	2.545	5.569
**3**	-6.8	3.741	6.490
**4**	-6.7	2.953	4.849
**5**	-6.7	4.317	7.301
**6**	-6.7	2.420	3.720
**7**	-6.6	5.933	9.131
**8**	-6.6	1.965	3.059
**9**	-6.5	4.129	7.032

Table S42. Score list of the molecular docking for hSTING-AQ-MK-1454.

Mode	Affinity (kcal/mol)	Dist from best mode	
		RMSD l.b.	RMSD u.b.
**1**	-7.3	0.000	0.000
**2**	-7.2	4.312	5.857
**3**	-7.1	4.017	10.104
**4**	-7.0	6.028	7.514
**5**	-6.8	5.926	9.750
**6**	-6.6	6.589	8.534
**7**	-6.4	5.658	8.890
**8**	-6.4	4.124	10.361
**9**	-6.3	7.375	10.156

Table S43. Score list of the molecular docking for hSTING-AQ-ribo/xylo-19.

Mode	Affinity (kcal/mol)	Dist from best mode	
		RMSD l.b.	RMSD u.b.
**1**	-7.9	0.000	0.000
**2**	-7.4	2.533	3.781
**3**	-7.3	2.885	8.776
**4**	-7.3	2.219	8.943
**5**	-7.3	7.246	9.775
**6**	-7.0	2.554	8.932
**7**	-6.7	3.362	6.452
**8**	-6.7	4.096	6.774
**9**	-6.5	1.764	2.387

Table S44. Score list of the molecular docking for hSTING-AQ-cyclic PNAs .

Mode	Affinity (kcal/mol)	Dist from best mode	
		RMSD l.b.	RMSD u.b.
1	-8.3	0.000	0.000
2	-8.2	1.961	2.397
3	-8.0	0.682	1.617
4	-8.0	3.108	10.921
5	-8.0	2.244	2.902
6	-7.8	2.115	2.937
7	-7.7	2.995	5.729
8	-7.7	3.331	10.418
9	-7.6	2.255	3.182

Table S45. Score list of the molecular docking for hSTING-AQ-DMXAA.

Mode	Affinity (kcal/mol)	Dist from best mode	
		RMSD l.b.	RMSD u.b.
1	-6.9	0.000	0.000
2	-6.8	1.506	4.877
3	-6.7	2.193	5.481
4	-6.3	1.485	4.714
5	-6.2	2.845	4.411
6	-6.2	3.349	6.197
7	-6.2	2.087	4.609
8	-6.2	2.151	6.505
9	-6.1	2.028	5.045

Table S46. Score list of the molecular docking for hSTING-AQ-α-Mangostin.

Mode	Affinity (kcal/mol)	Dist from best mode	
		RMSD l.b.	RMSD u.b.
1	-7.6	0.000	0.000
2	-7.5	2.219	4.106
3	-7.3	1.875	3.343
4	-6.9	1.607	2.237
5	-6.8	2.019	8.632
6	-6.6	2.260	8.394
7	-6.2	1.947	4.154
8	-6.2	2.225	4.093
9	-6.1	2.479	8.273

Table S47. Score list of the molecular docking for hSTING-AQ-di-ABZI.

Mode	Affinity (kcal/mol)	Dist from best mode	
		RMSD l.b.	RMSD u.b.
1	-6.3	0.000	0.000
2	-6.3	0.208	7.559
3	-6.1	2.670	5.776
4	-6.0	2.238	8.596
5	-6.0	2.256	4.925
6	-5.9	2.448	5.079
7	-5.8	6.345	10.291
8	-5.7	2.196	5.664
9	-5.6	2.712	5.654

Table S48. Score list of the molecular docking for hSTING-AQ-D61.

Mode	Affinity (kcal/mol)	Dist from best mode	
		RMSD l.b.	RMSD u.b.
1	-6.1	0.000	0.000
2	-6.1	1.890	5.822
3	-6.0	3.980	7.373
4	-5.9	3.226	6.793
5	-5.9	2.155	4.065
6	-5.9	3.535	4.828
7	-5.7	3.283	6.321
8	-5.7	2.478	7.236
9	-5.7	4.411	7.586

Table S49. Score list of the molecular docking for hSTING-HAQ-cGAMP.

Mode	Affinity (kcal/mol)	Dist from best mode	
		RMSD l.b.	RMSD u.b.
1	-7.4	0.000	0.000
2	-7.2	6.558	10.616
3	-7.0	1.030	2.056
4	-6.9	1.733	2.624
5	-6.9	4.415	9.561
6	-6.8	5.154	6.799
7	-6.6	6.979	9.856
8	-6.6	3.554	5.703
9	-6.5	2.969	4.604

Table S50. Score list of the molecular docking for hSTING-HAQ-c-AMP-CMP.

Mode	Affinity (kcal/mol)	Dist from best mode	
		RMSD l.b.	RMSD u.b.
1	-6.8	0.000	0.000
2	-6.7	2.609	3.092
3	-6.6	7.666	9.076
4	-6.5	1.453	1.533
5	-6.4	7.046	11.977
6	-6.3	6.888	8.882
7	-6.3	5.554	7.879
8	-6.2	2.180	2.640
9	-6.2	1.934	10.809

Table S51. Score list of the molecular docking for hSTING-HAQ-2′-F-c-di-GMP.

Mode	Affinity (kcal/mol)	Dist from best mode	
		RMSD l.b.	RMSD u.b.
1	-6.6	0.000	0.000
2	-6.6	0.221	5.811
3	-6.4	2.638	5.520
4	-6.4	2.616	3.694
5	-6.4	6.326	10.500
6	-6.4	6.334	10.334
7	-6.2	3.460	6.100
8	-6.2	3.456	7.272
9	-6.0	3.762	6.694

Table S52. Score list of the molecular docking for hSTING-HAQ-Rp, Rp-2',3-c-di-AMI.

Mode	Affinity (kcal/mol)	Dist from best mode	
		RMSD l.b.	RMSD u.b.
1	-6.9	0.000	0.000
2	-6.6	3.550	11.047
3	-6.6	7.850	10.773
4	-6.4	3.648	4.980
5	-6.4	2.700	10.697
6	-6.2	2.418	3.136
7	-6.1	7.327	10.656
8	-6.1	3.043	10.348
9	-6.0	2.537	3.408

Table S53. Score list of the molecular docking for hSTING-HAQ-CDG^{SF}.

Mode	Affinity (kcal/mol)	Dist from best mode	
		RMSD l.b.	RMSD u.b.
1	-6.8	0.000	0.000
2	-6.8	2.134	2.635
3	-6.5	1.894	5.995
4	-6.5	2.171	6.030
5	-6.3	8.077	11.217
6	-6.2	8.017	11.209
7	-6.2	9.345	12.800
8	-6.0	9.075	11.690
9	-6.0	9.253	11.765

Table S54. Score list of the molecular docking for hSTING-HAQ-MK-1454.

Mode	Affinity (kcal/mol)	Dist from best mode	
		RMSD l.b.	RMSD u.b.
1	-7.2	0.000	0.000
2	-7.1	4.324	5.871
3	-7.0	5.437	12.161
4	-6.9	6.055	7.531
5	-6.7	5.797	8.212
6	-6.7	3.997	10.130
7	-6.7	5.967	9.741
8	-6.7	6.670	8.615
9	-6.7	6.382	8.593

Table S55. Score list of the molecular docking for hSTING-HAQ-ribo/xylo-19.

Mode	Affinity (kcal/mol)	Dist from best mode	
		RMSD l.b.	RMSD u.b.
1	-7.3	0.000	0.000
2	-7.3	7.242	9.763
3	-7.3	2.328	8.989
4	-7.3	2.244	8.972
5	-7.2	2.527	3.764
6	-6.6	3.857	9.039
7	-6.6	6.255	10.197
8	-6.6	6.638	10.028
9	-6.5	6.589	9.197

Table S56. Score list of the molecular docking for hSTING-HAQ-cyclic PNAs .

Mode	Affinity (kcal/mol)	Dist from best mode	
		RMSD l.b.	RMSD u.b.
1	-8.3	0.000	0.000
2	-8.1	2.002	2.374
3	-7.9	3.199	10.548
4	-7.7	3.076	10.906
5	-7.6	3.231	10.646
6	-7.6	3.341	10.305
7	-7.5	2.114	8.333
8	-7.5	3.244	10.434
9	-7.4	2.276	3.235

Table S57. Score list of the molecular docking for hSTING-HAQ-DMXAA.

Mode	Affinity (kcal/mol)	Dist from best mode	
		RMSD l.b.	RMSD u.b.
**1**	-7.1	0.000	0.000
**2**	-7.1	1.485	4.877
**3**	-6.9	2.403	6.944
**4**	-6.8	2.035	3.015
**5**	-6.7	1.145	1.760
**6**	-6.6	1.665	3.736
**7**	-6.5	2.036	3.294
**8**	-6.4	1.895	4.317
**9**	-6.3	2.069	5.355

Table S58. Score list of the molecular docking for hSTING-HAQ-α-Mangostin.

Mode	Affinity (kcal/mol)	Dist from best mode	
		RMSD l.b.	RMSD u.b.
**1**	-7.5	0.000	0.000
**2**	-7.4	1.102	1.305
**3**	-7.4	2.227	4.111
**4**	-7.1	1.624	7.748
**5**	-7.0	2.033	8.596
**6**	-6.6	3.863	7.233
**7**	-6.5	2.250	4.015
**8**	-6.5	2.362	4.092
**9**	-6.4	4.081	9.645

Table S59. Score list of the molecular docking for hSTING-HAQ-di-ABZI.

Mode	Affinity (kcal/mol)	Dist from best mode	
		RMSD l.b.	RMSD u.b.
**1**	-6.3	0.000	0.000
**2**	-6.2	6.857	11.389
**3**	-6.2	0.687	1.308
**4**	-6.2	0.954	7.620
**5**	-6.1	2.610	4.856
**6**	-6.0	1.847	2.763
**7**	-5.9	2.275	4.914
**8**	-5.9	2.296	8.627
**9**	-5.6	2.612	7.519

Table S60. Score list of the molecular docking for hSTING-HAQ-D61.

Mode	Affinity (kcal/mol)	Dist from best mode	
		RMSD l.b.	RMSD u.b.
**1**	-6.0	0.000	0.000
**2**	-5.9	2.110	5.740
**3**	-5.9	3.508	4.751
**4**	-5.8	3.955	7.379
**5**	-5.7	2.241	4.221
**6**	-5.7	2.789	7.297
**7**	-5.5	3.223	6.967
**8**	-5.5	3.458	4.296
**9**	-5.4	3.149	7.154

Table S61. Molecular dynamics simulation RMSD value frequency distribution.

RMSD（nm）	hSTING-WT	hSTING-R232H	hSTING-R293Q	hSTING-AQ	hSTING-HAQ
**0**	0	0	0	0	0
**0.1**	1	2	2	1	2
**0.2**	5	6	6	10	6
**0.3**	27	27	17	9	8
**0.4**	39	24	21	9	59
**0.5**	60	70	14	30	45
**0.6**	57	105	6	39	53
**0.7**	74	83	41	90	56
**0.8**	32	138	81	238	122
**0.9**	71	283	24	117	84
**1**	202	438	72	70	146
**1.1**	277	465	57	174	784
**1.2**	769	294	29	763	959
**1.3**	1481	841	120	724	4114
**1.4**	1514	3016	250	1648	3563
**1.5**	2653	1310	169	4945	0
**1.6**	1863	2377	261	1134	0
**1.7**	835	521	245	0	0
**1.8**	41	1	203	0	0
**1.9**	0	0	5722	0	0
**2**	0	0	2661	0	0

Table S62. Mean and standard deviation analysis of the binding affinity of candidate agonists to different STING proteins.

Enzyme	Candidate agonists bind to different STING proteins （kcal/mol）	
	STANDARD DEVIATION	AVERAGE VALUE
**cGAMP**	0.164316767	-7.58
**c-AMP-CMP**	0.320936131	-7.36
**2′-F-c-di-GMP**	0.054772256	-6.64
**Rp, Rp-2',3-c-di-AMI**	0	-6.9
**CDG^{SF}**	0.258843582	-7.12
**MK-1454**	0.070710678	-7.2
**ribo/xylo-19**	0.328633535	-7.54
**cyclic PNAs**	0.089442719	-8.24
**DMXAA**	0.089442719	-6.94
**α-Mangostin**	0.130384048	-7.52
**di-ABZI**	0.216794834	-6.42
**D61**	0.151657509	-5.96

Figure S2.Results of the differential analysis of the binding energy of each small molecule agonist to 2', 3'-cGAMP agonists.

Dunnett's multiple comparisons test	Mean Diff.	95.00% CI of diff.	Significant?	Summary	Adjusted P Value	A-?	
cGAMP vs. c-AMP-CMP	-0.2200	-0.5559 to 0.1159	No	ns	0.3750	B	c-AMP-CMP
cGAMP vs. 2'-F-c-di-GMP	-0.9400	-1.276 to -0.6041	Yes	****	<0.0001	C	2'-F-c-di-GMP
cGAMP vs. Rp, Rp-2',3-c-di-AMI	-0.6800	-1.016 to -0.3441	Yes	****	<0.0001	D	Rp, Rp-2',3-c-di-AMI
cGAMP vs. CDGSF	-0.4600	-0.7959 to -0.1241	Yes	**	0.0028	E	CDGSF
cGAMP vs. MK-1454	-0.3800	-0.7159 to -0.04413	Yes	*	0.0192	F	MK-1454
cGAMP vs. ribo/xylo-19	-0.04000	-0.3759 to 0.2959	No	ns	0.9995	G	ribo/xylo-19
cGAMP vs. cyclic PNAs	0.6600	0.3241 to 0.9959	Yes	****	<0.0001	H	cyclic PNAs
cGAMP vs. DMXAA	-0.6400	-0.9759 to -0.3041	Yes	****	<0.0001	I	DMXAA
cGAMP vs. α-Mangostin	-0.06000	-0.3959 to 0.2759	No	ns	0.9993	J	α-Mangostin
cGAMP vs. di-ABZI	-1.160	-1.496 to -0.8241	Yes	****	<0.0001	K	di-ABZI
cGAMP vs. D61	-1.620	-1.956 to -1.284	Yes	****	<0.0001	L	D61
